# Neurotoxicity of Some Environmental Pollutants to Zebrafish

**DOI:** 10.3390/life14050640

**Published:** 2024-05-17

**Authors:** Teodora Maria Buzenchi Proca, Carmen Solcan, Gheorghe Solcan

**Affiliations:** 1 Department of Preclinics, Faculty of Veterinary Medicine, Iasi University of Life Sciences Ion Ionescu de la Brad, 700490 Iasi, Romania; buzenchi.teodora@yahoo.com (T.M.B.P.); csolcan@uaiasi.ro (C.S.); 2 Internal Medicine Unit, Clinics Department, Faculty of Veterinary Medicine, Iasi University of Life Sciences Ion Ionescu de la Brad, 700490 Iasi, Romania

**Keywords:** zebrafish, microplastics, fipronil, deltamethrin, rotenone

## Abstract

The aquatic environment encompasses a wide variety of pollutants, from plastics to drug residues, pesticides, food compounds, and other food by-products, and improper disposal of waste is the main cause of the accumulation of toxic substances in water. Monitoring, assessing, and attempting to control the effects of contaminants in the aquatic environment are necessary and essential to protect the environment and thus human and animal health, and the study of aquatic ecotoxicology has become topical. In this respect, zebrafish are used as model organisms to study the bioaccumulation, toxicity, and influence of environmental pollutants due to their structural, functional, and material advantages. There are many similarities between the metabolism and physiological structures of zebrafish and humans, and the nervous system structure, blood–brain barrier function, and social behavior of zebrafish are characteristics that make them an ideal animal model for studying neurotoxicity. The aim of the study was to highlight the neurotoxicity of nanoplastics, microplastics, fipronil, deltamethrin, and rotenone and to highlight the main behavioral, histological, and oxidative status changes produced in zebrafish exposed to them.

## 1. Introduction

Zebrafish (*Danio rerio*) were first mentioned in 1822 by the Scottish physicist Francis Hamilton, who identified them in the waters of the Kosi River in Bengal. Taxonomically, they are classified in the genus *Danio* and the family Cyprinidae; the genus includes about 44 species [1].

**Figure 1:**
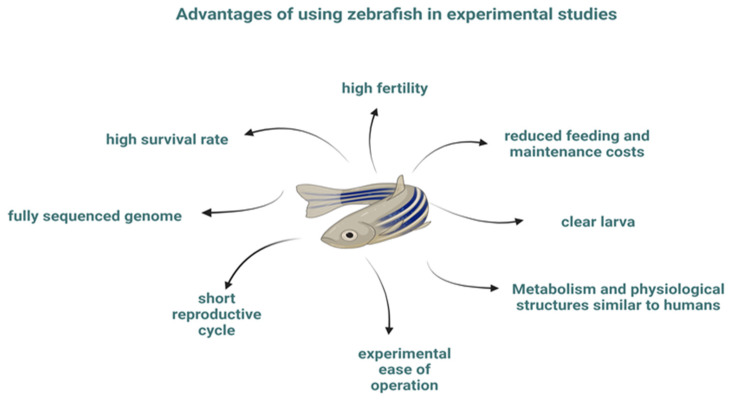
Schematic representation of advantages of using zebrafish in research and neuroscience. Created with www.BioRender.com (accessed 19 January 2024).

Zebrafish have become a viable alternative in studies aimed at investigating the toxicity of some pollutants, mainly due to their advantages (Figure 1), including a short reproductive cycle and high fecundity, rapid growth of their population (development from fertilized egg to adult takes about 3 months), ease of experimental operations and fully sequenced genome, high survival rate and low feeding and maintenance costs [2,3].

One of the most important advantages of zebrafish, however, is the transparency of their embryos and larvae, which allows direct manipulation and in vivo observation of the developmental processes of their internal structures and organs [4,5], as well as the study of accumulation sites of fluorescently labeled substances [5].

There are similarities between the metabolism and physiological structures of fish and humans, and in particular, the nervous system of zebrafish is similar to that of humans [6,7]. Fish exhibit a wide range of behavioral responses, such as social interaction, exploration, reproduction, and foraging, which change in response to environmental changes [8]. In addition, zebrafish have been shown to exhibit mammalian-like physiological responses and behavioral abnormalities as a result of being exposed to environmental pollutants, therefore they are widely used to assess the toxicity of some pollutants, but they are also used in many other areas, demonstrating their reliability. Therefore, the assessment of neurotoxicity triggered by some pollutants that are ubiquitous in the environment was the focus of this review, with the aim of presenting the particularities of the toxicants, the effects on the nervous system and behavior of exposed zebrafish, and the changes in oxidative stress enzymes and histological changes reported in the literature.

## 2. Nervous System of Zebrafish

In zebrafish, the patterning of nervous tissue in specific regions begins during gastrulation, and major brain segments, such as the forebrain (telencephalon, diencephalon), midbrain (midbrain), hindbrain (metencephalon) and spinal cord, are morphologically visible as early as one day post-fertilization [9,10]. Neuronal development is rapid and most adult neuronal cell types, neuronal circuits, and nuclei are functional at hatching and continue to develop in later larval stages [11].

At the time of hatching, zebrafish larvae exhibit complex behaviors and are able to respond differently to sensory stimuli [11]; for example, larvae are able to respond to threat, manifested as swimming rapidly away from the predator. More complex behaviors, such as social interactions and shoaling, will develop gradually in juvenile zebrafish over several weeks [12].

Zebrafish brain morphology and shape are typical for teleost fishes. The forebrain consists of the telencephalon and diencephalon. At the junction of the telencephalic and diencephalic tooth, the optic nerves and optic chiasm are readily visible from the ventral side and the habenula and pineal gland from the dorsal side [11]. The telencephalon consists of a dorsal structure forming two hemispheres that completely cover the ventral side, and the olfactory bulbs extend directly from the main telencephalic body [11]. The telencephalon also consists of two major parts, the dorsal pallium and ventral subpallium, each of which can be subdivided into smaller subregions based on morphological and functional differences [13]. The dorsal telencephalon (pallium) consists of several neuronal nuclei, and this dorsal part is thought to be equivalent to the mammalian neocortex and hippocampus, based on developmental origin, molecular markers and role in behavioral modulation [14,15]. The zebrafish pallium does not have a clear layered structure, as found in the mammalian neocortex, but exhibits distinct nuclear masses, which are thought to have a similar architecture to some of the basal circuits of the mammalian cortex. In addition, the interaction between multiple dorsal nuclei performs a similar function to the neocortex [11].

Diotel et al. [16] and O’Connell et al. [15] suggested that the lateral part of the dorsal telencephalon has hippocampal-like functions, and is involved in memory and spatial localization. The ventral telencephalon (subpallium) has a rostral part composed of four types of nuclei, forming a dorsal, ventral, central, and lateral part. Based on molecular markers, it was detected that the dorsal and ventral parts are equivalent to the basal ganglia and the dorsal part of the ventral segment is similar to the central amygdala [11]. The diencephalon consists of the preoptic region, habenula, posterior trabecula, pretectum, thalamic nuclei, and hypothalamus [11]. Based on connections and molecular markers, it has been described that the dorsal habenula of zebrafish is analogous to the mammalian medial habenula [17,18] and the ventral habenula to the mammalian lateral habenula [19]. The thalamic nuclei of zebrafish are located dorsally near the junction of the telencephalon and optic tectum, and although the arrangement and functions of the thalamus of zebrafish are similar to those of mammals, there are still differences, e.g., thalamo-cortical connections are missing in zebrafish [11,20]. Most of the diencephalon is represented by the hypothalamus, a structure responsible for controlling physiological homeostasis and behavior [11]. The hypothalamus controls endocrine hormones released by the pituitary gland, which controls stress response and growth [21], and connects the autonomic nervous system to other parts of the brain to control temperature, sleep, feeding, etc. [22].

Zebrafish also have a pineal gland, which, as in mammals, secretes the hormones indolamine and melatonin, as well as neurotransmitter subsets, which play key roles in regulating daily and seasonal rhythms [23].

The midbrain is dominated by the dorsally located large lobes of the optic tectum, which processes sensory information [11]. The optic tectum receives sensory information from the retina, and its primary function is to detect and process sensory stimuli and generate appropriate motor responses [24]. The midbrain is mainly represented by the cerebellum and its associated structures, which are easily distinguished macroscopically. The teleost cerebellum consists of a major lobe, the cerebellar body, and two bilateral lobes, which form the vestibulocerebellum [11]. Architecturally, the cerebellum of zebrafish is similar to that of mammals, but there is a significant difference between them, evidenced by the lack of deep cerebellar nuclei and well-defined white matter in zebrafish [11,25]. In addition, zebrafish exhibit a particular additional cell type in the cerebellar cortex, eurydendroid cells, which are thought to be equivalent to mammalian deep cerebellar nuclei [11]. The cerebellum has an important role in the integration of sensory information and motor control, as well as in cognitive functions [26], and the Mauthner neuron, part of specific neuronal groups in the hindbrain of zebrafish, has an important function in the escape response [12].

The peripheral nervous system of fish provides a link between the central nervous system and organs in the body, and is subdivided into the somatic nervous system (consisting of sensory and motor nerves) and the autonomic nervous system (divided into the enteric, sympathetic and parasympathetic nervous system, the latter of which innervates smooth muscles, skin, exocrine glands and internal organs) [11,27].

In zebrafish, the spinal cord has a mammal-like arrangement and shows areas of ventral and dorsal gray matter, equivalent to the dorsal and ventral medullary horns in mammals [11]. The spinal cord contains a wide variety of interneurons, sensory neurons, and motor neurons [28], and the interactions between different types of neurons control locomotor behaviors [29].

Neuronal proliferation in zebrafish is continuous, unlike in mammals, where it is limited in the adult brain [11]. Neurogenesis in zebrafish is spread along the entire rostro-caudal axis of the brain and spinal cord [30], and this constitutive generation of neurons relies on the presence of stem cells, located in niches in ventricular areas of the brain and spinal cord [31].

Recorded behaviors of zebrafish have correspondence with human perception, movement, and emotion [8], and their blood–brain barrier, which is similar to that of humans, has been well used for research aimed at screening for central nervous system drugs [32]. In addition, in behavioral neuroscience, zebrafish have been used as disease models for Parkinson’s disease (PD), Alzheimer’s disease (AD), and depression [33].

Thus, the nervous system and its composition, as well as the structure and function of the blood–brain barrier and the social behavior of zebrafish, are characteristics that make this small freshwater fish an ideal animal model for studying neurotoxicity [8].

## 3. Effects of Different-Sized Plastics on Zebrafish

Plastics are organic high-molecular-weight polymers that emanate from petroleum and include polyethylene, polypropylene, polyvinyl chloride and polyester, among which polyethylene and polypropylene rank first and second worldwide, respectively, followed by polyethylene terephthalate [34]. The emergence of plastics marked a revolutionary change in modern society due to their long-lasting corrosion resistance and ease of processing [35]. Since the first use of plastics in the 1950s, global plastic production has undergone substantial growth [36], and since the COVID-19 pandemic, there has been an acceleration of plastic pollution caused by improper storage of disposable materials used for personal protection (especially face masks).

The increase in world population and thus human activity has led to intensified agriculture, industrial development, and urbanization, all of which have contributed to a remarkable increase in waste production and massive environmental pollution [37,38]. Nowadays, plastic debris and fragment pollution have been recognized as a major water quality problem in both freshwater and marine systems [39]. In a study by Lacerda et al. [40] that exemplifies the issue of plastic pollution, they detected a concentration of 1794 pieces/km^2^ of plastic debris on the ocean surface in the Antarctic Peninsula. In addition, based on an analysis of statistical data collected between 2007 and 2013, it is estimated that in the future, at least 5.25 trillion plastic particles, totaling over 268,000 tons, will have been dumped in the ocean [41]. Coastal, marine, and riverine environments are under constant pressure from the anthropogenic release of pesticides, heavy metals, hydrocarbons, persistent organic pollutants, flame retardants and organophosphorus substances, pharmaceuticals, personal care products and, especially, plastics into the environment [42,43,44,45,46]. As plastics enter the aquatic environment, they undergo photodegradation and alteration, fragmenting into smaller particles ranging in size from nanometers to micrometers [47]. This fragmentation leads to the formation of microplastics and nanoplastics in aquatic ecosystems, which pose a significant ecological threat [48], affecting the behavior and reproductive health of aquatic organisms and endangering human health [49].

### 3.1. Effects of Exposure to Nanoplastics in Zebrafish

Nanoplastics are plastic particles produced and released into the environment as a result of improper waste management and excessive industrial use of plastics [50,51]. Attempts to define nanoplastics have sparked controversy among researchers, mainly due to differences of opinion regarding their size, but the definition of nanoplastics as particles that are produced unintentionally (either through the manufacture of plastic products or their degradation) and are in a colloidal state, with size varying in the range of 1–100 nm [5,50,52], is the relevant one for the current state of knowledge. Nanoplastics are widespread in aquatic ecosystems due to sewage discharge, aerial deposition, spills, and runoff [53] and can be divided into two groups: (a) natural nanoplastics (e.g., aerosols, desert dust, emissions from volcanic activity, etc.) and (b) anthropogenic (e.g., from metal oxides, fossil fuel combustion, vehicle exhaust emissions, drug production, emissions from mining demolitions, etc.) [53,54,55]. The most prevalent type of nanoplastic in the aquatic environment is titanium dioxide (TiO_2_), which is reported by the European Union to be one of the main pollutants of surface water, with a quantitative proportion of 2.2 µg/L [54].

Nanoplastics in the aquatic system originate from primary and secondary particles; primary particles are the result of intentional manufacture according to a standard pre-determined size, while secondary particles are derived from the fragmentation of larger particles [56]. Nanofragmentation can involve one of two mechanisms [39]: a major process in which the nanofragmentation occurs at the surface of macro- and microplastics, or a minor process, in which further gradual size reductions occur, produced by the degradation phenomenon.

Degradation of plastics can be induced chemically, physically, or biologically. Six processes involved in the degradation of plastics have been described: thermal degradation, hydrolysis, thermo-oxidative degradation, photodegradation, biodegradation, and mechanical or physical degradation [57]. Thermal degradation can be overlooked when talking about the aquatic environment, as plastics are subjected to moderate temperatures at this level, and temperatures up to 430 °C are used in factories to induce the decomposition of plastics [58]. Mechanical degradation is most common in aquatic environments and occurs due to mechanical stress from waves, sand, rocks, and other forces or substances, which create interactions with the polymers in water. When plastic is subjected to mechanical shear force, the molecules break down [56]. In addition, there are also biodegradable plastics, which can be broken down in water by microorganisms in the habitat such as fungi and bacteria [56]. However, in a general sense, the most important nanofragmentation process results from embrittlement, followed by physical abrasion of microplastics [39]. In addition to industrial operations and human activities leading to the accumulation of plastic waste, environmental phenomena can also be sources of nanoplastics, such as tsunamis or storms, which can contribute significantly to the spread of plastic particles [59]. The main source of a significant amount of nanoplastic production is cosmetics [60], which can end up in the aquatic environment via wastewater or during consumer use [61]. Other products categorized as sources of nanoplastics include water-based paints, adhesives, biomedical products, pharmaceuticals, and electronic and magnetic products. Zhang et al. [62] demonstrated that thermal cutting of polystyrene foam emits nanosized particles (approx. 22–220 nm), and Stephens et al. [63] demonstrated that 3D printing also emits considerable amounts of plastic nanoparticles (approx. 11–116 nm).

At present, nanoplastics are the least known type of aquatic waste, yet are considered potentially the most hazardous [39]. Plastic materials known to be nontoxic at the macroscale have been shown to acquire toxicity at the nanoscale [64], which potentiates their adverse effects in the environment, and it has been reported that the atoms on the surface of nanoplastics have unsaturated bonds, which give them higher reactivity than the material they come from, also potentiating the toxic effect [65,66].

Nanoplastics have a larger surface area than macroscale materials, and this is an important parameter when considering the toxic effect [56]. Practically, the large surface area of nanoplastics implies that it retains certain toxic chemicals, which can increase the overall hazard of plastic nanoparticles [39]. Their large surface area, and thus their hydrophobicity, generates additional toxic effects, in that they become able to bind to other environmental contaminants [52,67,68].

Observations of nanoplastic ingestion by aquatic organisms have directed research toward unraveling the role of nanoplastics as a vector for other toxic compounds [50], since marine animals, especially fish, are exposed to a mixture of contaminants, which makes it necessary to understand the potential function of nanoplastics in the bioavailability and biological effects generated by other adsorbent organic pollutants [69,70]. An experimental study conducted by Barreto et al. [69], in which zebrafish were exposed to simvastatin (a hypolipidemic drug) and nanoplastics in different concentrations, was aimed at highlighting the effects generated by these substances administered individually and in combination. The results of the experiment showed that the combined effect was less toxic than the individual effect induced by each substance separately. However, synergistic effects were also identified, especially in terms of fish survival rate (mortality of organisms was recorded less than 48 h after exposure, when the highest concentrations were used), but also additive effects, evidenced by the occurrence of malformations [69]. Basically, this experimental study demonstrated that nanoplastics can modulate the toxicity of simvastatin to zebrafish embryos and larvae, with less pronounced or similar effects of the drug when associated with nanoplastics.

The effects of nanoplastics on the toxicity of other contaminants depend on the exposure concentrations: the higher the concentration of nanoplastics, the higher the toxicity. Limitations associated with analytical methods make it impossible to quantify nanoplastics in the environment [39,67]. However, there are increasing levels of nanoplastics in the aquatic environment, and this increase is caused by their continuous release and consistent degradation [5,71,72]. The accumulation of large amounts of nanoplastics in the aquatic environment poses a serious threat to the health of both invertebrates and vertebrates in that habitat and can indirectly but markedly (via the food chain) influence human health.

Nanoplastics in water are in a colloidal state, which makes them more reactive and toxic than microplastics, especially as their small size facilitates ingestion by marine animals and allows them to penetrate biological barriers. So far, nanoplastics have been shown to pass from algae to plankton and fish [73,74,75,76] and to interact with living organisms as either plastic nanoparticles or aggregates [56]. Nanoplastics are readily absorbed by fish, either directly by ingestion or through the gills, and have been detected in the blood circulation of zebrafish embryos (96 h post fecundation (hpf)), with subsequent gradual accumulation in the liver and digestive tract. Nanoplastics were detected in the brain and eyes of zebrafish larvae at 120 hpf, proving their ability to penetrate the blood–brain barrier [77,78]. Pitt et al. [5] and Parenti et al. [72] demonstrated that polystyrene nanoparticles can penetrate the chorion and accumulate in zebrafish embryo tissue, triggering bradycardia and hyporesponsiveness. Bioaccumulation of nanoplastics has also been identified in the brain, gills, blood, liver, and digestive tract of zebrafish immediately after hatching and has been shown to cause oxidative DNA damage and developmental malformations, among other problems [79]. Fluorescence spectroscopic analysis of tissues treated with polystyrene nanoplastics showed strong green fluorescence, observed predominantly in the gonads, intestine, liver, and brain, but with a significant proportion in the gonads (about five times more than in the brain) [49].

The primary abnormalities that fish exhibit after being exposed to nanoplastics are neurobehavioral in nature [49], and in most experimental studies, zebrafish have been exposed to polystyrene nanoplastics (PS-NPs) to assess the toxic effects. Exposure to PS-NPs was found to alter behavioral parameters, decreasing the fishes’ interest in exploration; they exhibited stay-at-the-bottom behavior in the aquarium they were introduced to, with no improvement in anxiety as they adapted to the new environment, and at the highest concentration of exposure, they exhibited hyperreactivity-like behavior, highlighted by increased mean velocity [80]. The state of fear, manifested by anxious or even freezing behavior, although an innate response that is automatically generated when fish are confronted with natural predators, is altered after exposure to nanoplastics, which disrupts the response to predators. Fish in the treatment group were found to maintain a small average distance between themselves and predator fish, and there was even a slight increase in the time to approach predators. Basically, PS-NPs reduced the performance of predator avoidance behavior [80].

Social interaction was found to not change after acute exposure regardless of nanoplastic concentration. Shoaling behavior, an innate behavior specific to zebrafish that involves grouping and swimming in schools to reduce anxiety and the risk of being captured by predators, was enhanced after exposure to PS-NPs. Both concentrations tested triggered overt shoaling behavior in exposed fish.

Concerning circadian rhythm, reduced locomotor activity and abnormal movement orientation were observed in fish in treatment groups during the light cycle, highlighted by decreases in mean velocity and the movement–frozen ratio, while during the dark cycle, hypoactive behavior was evident, evidenced by decreased mean velocity, mean angular velocity and movement–frozen ratio in exposed fish compared to the control group. Chronic exposure to PS-NPs reduced aggression and predator avoidance in zebrafish and led to decreased social motivation and intention to interact with social partners [80]. Reduced locomotion behavior was also observed, with decreased mean speed and increased freezing time.

Biochemical measurement of neurotransmitters in the brain by enzyme-linked immunosorbent assay (ELISA) after exposure to PS-NPs showed inhibition of acetylcholinesterase activity (AChE) and decreased levels of dopamine, melatonin, Γ-aminobutyric acid (GABA), serotonin, vasopressin and oxytocin. Prolactin and vasotocin did not show appreciable changes in fish in the treatment groups [80]. Although AChE was decreased, the amount of acetylcholine did not change in both treatment groups, only in the group exposed to the highest concentration of nanoplastics, suggesting that AChE inhibition is exposure dose-dependent. Given the altered levels of neurotransmitters, a connection can be made between the recorded abnormal behaviors and the dysregulation of the mentioned biomarkers.

Oxytocin, a hormone responsible for dampening the stress and anxiety response and modulating a variety of behaviors, and vasopressin showed low post-exposure levels, which also explains the occurrence of abnormal behaviors such as decreased exploratory behavior and reduced aggression in zebrafish [80]. Also, decreased AChE activity may contribute to triggering anxiety-like behaviors, followed by increased central nervous system-related glial fibrillary acidic protein (GFAP) and alpha-tubulin genes [50]. Decreased GABA may be responsible for the abnormal fear-related response contributing to the decreased predator avoidance behavior identified in fish in the treatment groups [80]. Changes in circadian rhythm were not attributed to changes in melatonin levels in the fish but to anxiety and reduced vasopressin levels.

Lu et al. [81] exposed zebrafish larvae and embryos to PS-NPs for 120 h and reported that nanoparticles penetrating the embryonic brain induced neuronal loss and interfered with the GABA-ergic, cholinergic and serotonergic systems in particular, affecting neuronal signaling and generating behavioral abnormalities. Also, in a study by Lee et al. [52] when zebrafish embryos were exposed to fluorescent plastic nanoparticles combined with gold ions, there was an increased mortality rate and activation of the inflammatory response, evidenced by increased expression of interleukins IL-6 and IL-1β. Teng et al. [82] exposed zebrafish larvae to PS-NPs for 120 h and observed an increase in apoptosis in the brain along with neurobehavioral impairment as well as decreased glycine, cysteine, glutathione, and glutamate levels. Chen et al. [83] exposed zebrafish to nanoplastics and bisphenol A (the most widely used additive in polycarbonate plastics) and demonstrated that bisphenol A accumulated in the organs of zebrafish and was responsible for neurotoxic effects on the central nervous and dopaminergic systems. In this regard, they reported increased myelin basic protein and tubulin genes in the central nervous system, increased expression of astrocyte-derived neurotrophic factor in the midbrain, and significant inhibition of AChE [83].

In conclusion, nanoplastics can generate behavioral impairments and neurotransmitter alterations after both acute and chronic exposure in both embryos/larvae and adult fish.

Nanoplastics have also been shown to affect redox homeostasis and mitochondrial well-being, acting at multiple levels and in multiple tissues, directly or as vectors for other pollutants [84], and to be responsible for the development of oxidative stress. Exposure to PS-NPs generates oxidative stress through the overproduction of reactive oxygen species (ROS) and a significant decrease in superoxide dismutase (SOD). Increased catalase (CAT) and glutathione peroxidase (GPx) levels and significantly decreased adenosine triphosphate (ATP) levels were also observed [80]. Other experimental studies detected reduced CAT activity, leading to oxidative damage, which interfered with the glutaminergic and cholinergic neurotransmitter system and generated apoptosis of brain cells [76,85,86]. Thus, CAT and SOD levels are parameters that vary, with either lower or higher values depending on the concentration of nanoplastics and the age of the exposed fish.

### 3.2. Effects of Exposure to Microplastics in Zebrafish

Microplastics are synthetic solid particles or polymer matrices that are insoluble in water, range in size from 1 µm to 5 mm, and can be of primary or secondary origin [87]. They can be found in the form of fibers, film, foam, spheres, or pellets [88]. Agreement on the upper size limit of microplastics (5 mm) is consistent in the literature [89], but studies have reported different lower limits [90,91]. Thompson et al. [92] described 20 µm sized particles accumulating in the aquatic system as microplastics. Standardization of the size range of microplastics and agreement on particle subclassifications are necessary because as particles shrink, they acquire different properties relative to the material from which they originated that can influence their environmental fate and how much they spread [93]. In addition, very small sizes can amplify the adverse effects on exposed organisms [94], as is noted for nanoplastics.

In the environment, microplastics are classified as primary or secondary, differentiated according to their source of origin. Primary microplastics are those that are used intentionally, such as in cosmetics, scrubs, and shampoos, or as particles for blasting or resin granules for pre-production [95]. Secondary microplastics are the result of the fragmentation of larger plastics, either through actual use (e.g., fibers from washing clothes), inadequate waste management, or the degradative action of environmental factors, which affects plastic bags and bottles [91], the most common macroplastics found in the environment. To date, most research has focused on primary microplastics [96], as the rate of formation of secondary microplastics is thought to be influenced by several factors, such as polymer type and environmental conditions [97], making it difficult to accurately estimate the quantity of their emission. It is known, however, that the fragmentation of plastics is highly dependent on temperature and the amount of UV radiation [98,99], as well as on the mechanical action of waves and currents (in the aquatic environment) or microbial degradation, although microplastics are considered to be resistant to biotic degradation [100]. Gradual fragmentation of microplastics will lead to the formation of nanoplastics, with enhanced toxic properties.

The sources of microplastic generation are many and varied and include, among others, plastic product manufacturing [101], wastewater treatment plants [102], industrial and agricultural waste [103,104], degradation of plastics in the environment [105] and fisheries and aquaculture [106,107], all of which can facilitate the entry of microplastics into aquatic systems and, of course, affect organisms in the habitat [108,109,110]. It is considered that the sources of microplastics can be divided into four types: large plastic debris, medicines, textiles, cosmetics, and cleaning products [84]. From these sources, they can easily enter aquatic environments, mostly via sewage treatment plants, and then spread worldwide via wind and currents [98,111,112].

Skincare products and synthetic clothing are important sources of microplastic generation. Skincare products are transported through sewage systems along with wastewater [113] and subsequently accumulate in aquatic ecosystems [100], and synthetic clothing contains microplastics in the form of fibers, with about 700,000 fibers released from 6 kg of clothing in a single wash, on average [114]. Plastic pellets used as raw materials in various industrial applications are also released into the environment [115], as well as microplastics used in dental brackets and in the pharmaceutical industry, which reach the environment via wastewater [100]. Sundt et al. [116] assessed secondary and primary microplastic emissions in Norway and reported that tire dust is the largest contributor to microplastic concentrations in the Baltic Sea, while cosmetics contribute the least. Lassen et al. [91] obtained similar results, reporting that tire dust contributes 60% of the total microplastic emissions to the aquatic environment, while cosmetics contribute 0.1%. Recognizing the source of microplastic generation is important in order to conduct a correct and accurate assessment of the amount of microplastics entering the aquatic environment and institute safe and favorable measures to mitigate the imminent risks associated with them [100]. It is also necessary to know the distribution of microplastics, as the impact is greater where microplastics accumulate than where they exist in small quantities.

Microplastics have been identified in most species at all phases of the marine food chain [117], and it is well known that aquatic organisms ingest microplastics with their food due to their similar size [118,119]. In addition, microplastics have been shown to accumulate in zooplankton, marine worms, mussels, crabs, and fish [73,74,75,76]. Marn et al. [120] reported that nearly 700 aquatic species worldwide have been adversely affected by microplastics, including sea turtles, penguins, and crustaceans. Higher-density microplastics are biologically available to benthic species, while lower-density microplastics are available mainly to pelagic species [109]. The composition of microplastics is therefore important; for example, polypropylene and polyethylene have a low density and create debris that is less dense than water, which ensures that they float, while polyethylene terephthalate, polystyrene and cellulose acetate create debris that is more dense than water and therefore sinks [121]. Microplastics with positive buoyancy will float on the water surface only temporarily, as dirt accumulates on their surface and they will, over time, reach the benthic zone [100].

The small size of microplastics means they are ingested by aquatic species, which disrupts their physiological systems, and they subsequently move up the food web, causing health problems in humans [100], as humans are the ultimate consumers of marine food [122]. Recent studies have confirmed the presence of microplastics in human stool [106] and placenta [123], which is of particular concern.

Microplastics are generally rapidly absorbed and excreted by many marine species, which does not preclude the existence of conclusive evidence of biomagnification [124]. Practically, the ingestion of microplastics must also be considered in direct relation to digestion rates in order to make a correct interpretation of their presence in the bodies of aquatic species [89]. As with nanoplastics, it has been reported that due to their physicochemical properties, microplastics adsorb significant amounts of hydrophobic organic contaminants and that after being ingested by aquatic species, they can act as vectors for the transport of organic contaminants into the body [73,125]. It has also been shown that fragmentation of microplastics can release hazardous organic pollutants such as dichlorodiphenyl trichloroethane, diethyl ethers, polybrominated ethers, and other additives that are incorporated during manufacture, thereby potentiating their toxicity by increasing their concentration [126,127,128,129,130]. In addition, microplastics can accumulate metals, inorganic contaminants, and organic chemicals from the surrounding environment [100,131], and the uptake of these microplastics into the bodies of marine animals results in increased toxicity due to the aggregation of hydrophobic organic compounds [132].

Numerous studies have reported the biological toxicity of microplastics to aquatic organisms, as evidenced by neurotoxicity, developmental and reproductive toxicity, immunotoxicity, genotoxicity and the potential for transgenerational transmission [133,134,135], and the effects of microplastic ingestion have been shown to include reduced dietary intake, developmental disorders and behavioral changes [100]. Three forms of detrimental effects related to the ingestion and uptake of microplastics in marine species are considered [100]:

Regarding the physiological effects attributed to ingestion, it has been shown that the greater the number of microplastics ingested, the more likely the risk of developmental disturbance [136,137,138].

Fatal reactions can occur, caused by the release of compounds incorporated during plastic manufacture (such as additives, plasticizers, antioxidants, flame retardants, pigments, etc.), which can leach into tissues, leading to bioaccumulation [100].

There can be triggering of additional harmful reactions due to the unintentional ablation by microplastics of pollutants (polycyclic aromatic hydrocarbons, organochlorine pesticides, benzenes, hexachlorocyclohexanes, etc.), or heavy metals (chromium, copper, lead, cadmium, etc.) [49,100,139].

In zebrafish, microplastics tend to accumulate in the gills, liver, and intestine. Their transport through the fish’s organelles is facilitated by the circulatory system, and the caliber of the blood vessels plays an important role in this respect [140]. Microplastics usually accumulate in adult zebrafish and less frequently in embryos and larvae. Short- or long-term exposure to microplastics has negative effects on their nervous system and can lead to behavioral abnormalities, which can manifest as significant changes in swimming, social interaction, and reproductive behaviors, including impaired fertility [49,80]. Current studies suggest the existence of several mechanisms responsible for microplastic toxicity in zebrafish, but the most relevant is considered to be oxidative stress, which can interfere with normal nervous system function and energy metabolism [50,141]. Microplastics have been shown to cause oxidative damage in specific regions of the brain, posing a risk to development, including neurodevelopment [8,142], which can lead to an imbalance between ROS production and the antioxidant capacity to scavenge them [49], thus maintaining ongoing oxidative stress. For example, zebrafish exposed to polystyrene microplastics had significantly increased ROS levels and altered CAT, SOD and GPx expression [5]. Basically, polystyrene microplastics were found to trigger a significant increase in SOD and CAT activity [81]; these enzymes generally play an important role in mitigating the negative effects generated by ROS overproduction [143]. Excess ROS accumulation can interfere with nervous system mechanisms, and due to oxidative stress, neurons can suffer oxidative damage and thus undergo apoptosis, which accelerates the degenerative process and triggers neuroinflammation, which in turn affects the normal morphology and function of neurons [144].

Behavioral disturbances have also been identified after zebrafish were exposed to microplastics, which are thought to be caused by the consequent occurrence of oxidative stress [49]. Microplastics can penetrate into and accumulate in the zebrafish brain, and at that level can trigger the overproduction of ROS, leading to behavioral disturbances and brain damage [145]. Yu et al. [146] exposed zebrafish to polystyrene microplastics and reported the onset of anxiety-like behaviors and altered motor skills with increased swimming distance. Similarly, Mak et al. [147] exposed adult zebrafish to polyethylene microplastics and reported that the fish exhibited erratic movements and epileptic-like behavior, characterized by tail bending down or up. Zebrafish larvae exposed to a microplastic mixture for 14 days showed reduced mean swimming speed and total distance traveled, as well as impaired predator avoidance behavior and response to aversive stimuli [125]. In addition, it has been shown that exposure to microplastics can alter the expression of genes in fish related to neurodevelopment and neurotransmission, acting by altering the levels of neurotransmitters such as dopamine and acetylcholine, leading to neurotoxicity [145,148]. For example, polystyrene microplastics decreased the movement distance of zebrafish larvae in response to exogenous dopamine, indicating that abnormal dopamine levels affect animal behavior, and similar effects were observed in exposed adult zebrafish, which showed impaired motor behavior and altered AChE gene activity and expression [50]. Histologically, fish exposed to polystyrene microplastics showed pathological changes in brain tissue, including inflammatory cell infiltration, cytoplasmic vacuolization, degeneration, and neuronal death [149].

The combined effects of microplastics and other contaminants are synergistic or additive, enhancing bioaccumulation and toxicity [150]. A conclusive example is related to heavy metals: microplastics were found to increase the degree of cadmium-induced oxidative damage and inflammation in zebrafish tissues, affecting social behavior and influencing reproductive success [49,151]. Also, the interaction of microplastics with persistent organic pollutants alters sex hormone levels in fish and facilitates accumulation in gonadal tissue [152].

A particular case involved exposing zebrafish to methionine, polypropylene microplastics and the combination of both, where it was shown that the two toxicants did not act synergistically, and it was concluded that microplastics are able to block the toxic effects of methionine [86].

In conclusion, swimming ability and vitality for defense, predation, and mating are impaired after zebrafish are exposed to microplastics, and levels of oxidative stress enzymes and neurotransmitters are affected. However, the oxidative stress and impaired redox homeostasis induced by microplastics are influenced by the age of the fish, as well as the time of exposure and concentration of microplastics. Clearly, short-term exposure at low concentrations in the early life stage of zebrafish (4–96 hpf) will result in only weak toxic effects, whereas long-term exposure will lead to neurotoxicity, impaired swimming behavior, and reduced body weight, with these effects subsequently being transmitted to offspring [153].

In terms of the effects generated, both nanoplastics and microplastics can exert neurotoxic effects on zebrafish due to their ability to interact with the nervous system through various mechanisms. Plastic particles can have a neurological impact on zebrafish, and the following mechanisms are relevant:

- Ingestion and accumulation: Zebrafish, like many aquatic organisms, can ingest microplastics and nanoplastics either directly or indirectly through the food chain. Once ingested, these particles can accumulate in various tissues, including the brain and nervous system.
- Physical damage: Microplastics and nanoplastics can cause physical damage to the nervous system of zebrafish. These particles can disrupt neuronal connections, interfere with synaptic transmission, and induce inflammation in brain tissues. Accumulation of plastic particles in neuronal tissues can lead to structural abnormalities and impaired neuronal function.
- Leaching of chemicals: Nanoplastics and microplastics can release adsorbed chemical additives and pollutants into the environment, including the water column and sediments. These chemicals include neurotoxic substances such as plasticizers, flame retardants, and persistent organic pollutants (POPs). Once released, these neurotoxic chemicals can be absorbed by zebrafish and affect the function of their nervous system.
- Oxidative stress and neuroinflammation: Exposure to microplastics and nanoplastics can induce oxidative stress and neuroinflammation in zebrafish. The presence of plastic particles in neuronal tissues can trigger the production of ROS and inflammatory mediators, leading to cell damage and dysfunction within the nervous system. Oxidative stress and neuroinflammation can disrupt neuronal signaling pathways and contribute to neurobehavioral abnormalities in zebrafish.
- Behavioral and cognitive effects: Neurotoxic effects induced by microplastics and nanoplastics can manifest as altered behavior and cognitive function in zebrafish. Studies have shown that exposure to plastic particles can affect locomotor activity, learning and memory, social behavior, and predator avoidance responses in zebrafish. These behavioral changes can result from direct neurotoxicity or the secondary effects of neuronal damage and dysfunction caused by plastic exposure.

In general, nanoplastics and microplastics pose significant neurotoxic risks to zebrafish by disrupting neuronal function, inducing oxidative stress and inflammation, and altering behavior and cognitive ability. Understanding these neurotoxic effects is essential to assess the ecological impact of plastic pollution on aquatic organisms and to implement effective mitigation strategies to protect aquatic ecosystems.

## 4. Neurotoxicity of Fipronil to Zebrafish

Insecticides are used all over the world and are the main source of chemical contamination, as they are misused in agricultural and urban areas. Indirectly, they affect the structure and composition of water and soil and therefore the organisms in these habitats, mainly because of the remarkable resistance of these substances in the environment [154], and they pose a danger to aquatic organisms, especially in freshwater ecosystems [155]. Organochlorines, organophosphates, carbamates, pyrethroids, neonicotinoids, and fiproles are the best-known classes of insecticides [156,157], but at the top of the list in terms of use today are neonicotinoids and fiproles [158]. Fipronil (FIP), the first phenyl pyrazole insecticide to be widely used for pest control, is known to cause neurotoxicity via interactions with GABA and glutamate receptors, although alternative mechanisms have been described [159]. Sources of fipronil pollution can result from different waste streams [159]: from its use as an insecticide and from its use as a veterinary ectoparasiticide. These lead to direct release into the environment, and even if not applied near surface water, phenyl pyrazole compounds bind to soil organics, are transported by migration and elution processes, and accumulate in urban freshwater systems [160]. This is the main reason why fipronil has been detected in surface water and sediment.

**Figure 2:**
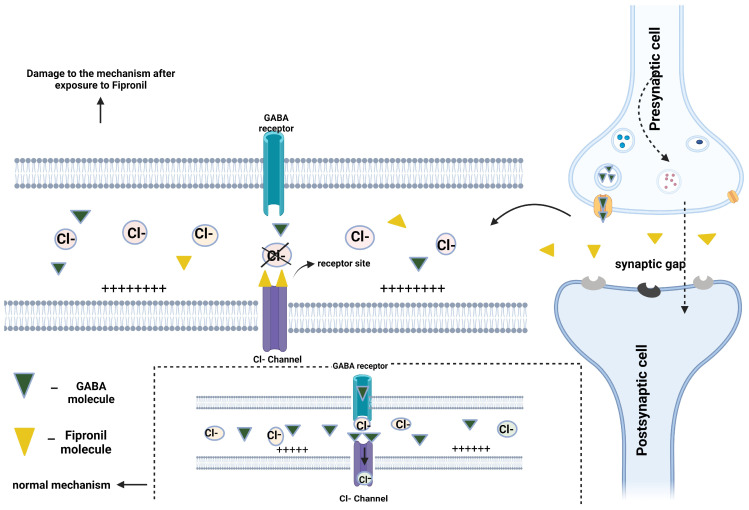
Fipronil disrupts chloride channels in insect cell membranes. Basically, binding of insecticide molecules to specific chloride channel sites leads to inhibition of neurotransmitters GABA and glutamate. This results in hyperexcitation of the central nervous system. Figure was created with www.BioRender.com (accessed 19 January 2024).

Insecticides, in general, act on the nervous system of insects, leading to abnormal functioning of neurotransmitters. Basically, insecticide molecules bind at neurotransmitter sites and deregulate the functions of specific cellular channels (Figure 2) [161]. FIP is characterized by the disruption of chloride channels in insect cell membranes [161,162], and in addition to affecting chloride channels in the nervous system, it can also affect those in the muscles or kidneys [161]. The mechanism of action of FIP is illustrated in Figure 2.

FIP is known to block the passage of chloride ions through GABA-regulated chloride channels, disrupting central nervous system activity. Organic nitriles decompose to cyanide ions both in vivo and in vitro, therefore the main mechanism of toxicity for organic nitrites is the production of toxic cyanide ions or hydrogen cyanide. Cyanide is an inhibitor of cytochrome c oxidase, of the fourth electron transport chain complex, and it complexes with the ferric iron atoms in this enzyme. Cyanide binding to this cytochrome prevents electron transport from cytochrome c oxidase to oxygen. As a result, the electron transport chain is interrupted and the cell can no longer produce aerobic ATP for energy, and tissues that depend mainly on aerobic respiration, such as those in the central nervous system and the heart, are particularly affected. In addition, cyanide is known to bind to the ferric ion of methemoglobin to form inactive cyanohemoglobin [159]. FIP also has an inhibitory effect on glycine receptors in animals, especially small aquatic animals [163]. Glycine is a neurotransmitter that can play a role in inhibiting membrane potential depolarization and in temporarily maintaining an elevated membrane potential, called excitatory postsynaptic potential. Thus, when fipronil binds to glycine receptors, excitatory postsynaptic potential is produced in the central nervous system, leading to muscle convulsions, spasms, and respiratory muscle injury [164]. In 2013, FIP was banned in Europe for agricultural applications [165], but studying it is still relevant as it has started to be used in antiparasitic treatments for dogs and cats (by topical administration) with control action against ticks, ear mites and fleas [166,167,168]. Exposure of humans to FIP through accidental contact, either in an acute or chronic event or a suicide attempt [168,169], has been found to trigger a range of negative consequences, most notably cytotoxicity, neurotoxicity, hepatotoxicity, and reproductive problems [158].

FIP can be degraded by light, water or soil microorganisms, and the main resulting degradation product is fipronil sulfide [156,157,160,161,170,171]. The degradation products have much higher resistance and persistence in the environment under both aerobic and anaerobic conditions, and these characteristics confer a much higher degree of toxicity to the metabolites of FIP than to the substance itself [172]. In addition, FIP persists for a long time in aquatic habitats, mainly because of its low solubility, and has been shown to be lethal to aquatic organisms [160,173,174]. In the aquatic environment, FIP is converted by photolysis to fipronil-desulfinyl, which is more readily taken up by organisms and thus is more toxic [160]. After insects, aquatic organisms are the next target of FIP, and lethal doses for them are very low [160]. Several laboratory studies on the acute sublethal effects of FIP have reported toxic effects in zebrafish embryos, including developmental toxicity [175], endocrine disruption [176] and behavioral impairment [177,178]. In addition, FIP has been shown to be involved in altering DNA methylation in zebrafish embryos [179]. Robea et al. [158] studied both FIP and pyriproxyfen (PYR), which is an insecticide that affects insect activity by mimicking natural hormones involved in maturation. Basically, PYR is an endocrine disruptor that mimics the juvenile hormone required for insects to progress from the immature to the adult stage of development [180]. PYR is characterized by low solubility and hydrophobicity, which indicates its ability to persist in the environment, therefore precautionary measures must be implemented regarding its use [180,181].

Although PYR is used for insect control, it has been shown to be a nontoxic compound, and in 2006, the World Health Organization (WHO) recommended using it (at a dose of 0.01 mg/L) to disinfect drinking water, specifically to combat Dengue fever. Subsequently, in 2014, the Brazilian Ministry of Health established the same recommendations for use, but with regard to mosquito control [182]. Recent research on mice, rats, and zebrafish has proven the opposite in terms of PYR’s lack of toxicity. PYR has been shown to be as toxic as other insecticides, causing impaired AChE activity, oxidative stress, and malfunction of calcium ion transport in zebrafish [183].

A high percentage of insecticides in current use are considered to be potentially neurotoxic, affecting the nervous system of exposed organisms [158]. In fish with prolonged exposure to FIP, hyperreactivity can be initiated and maintained [158]; this disturbance is due to the accumulation of FIP in the body, which has a remarkable effect on the neurotransmitter GABA, responsible for triggering behavioral changes.

Oxidative stress parameters after exposure of zebrafish to FIP + PYR showed the following changes [158]:

- Increased SOD levels.
- Increased lipid peroxidation (GPx) levels.
- Increased malondialdehyde (MDA) levels.

Studied separately, different substances generate different effects. FIP, analyzed as a separate entity, was found to cause increased SOD and CAT activity and increased lipid peroxidation levels in brain and kidney and to trigger neurotoxic effects in the brain of zebrafish embryos, larvae and adults, while PYR caused decreased SOD, CAT and lipid peroxidation levels. The main effects of zebrafish exposure to PYR are summarized in Table 1. The effects of PYR are listed in Table 2.

In fish exposed to the PYR + FIP mixture, higher values were recorded for total distance traveled during all behavioral testing sessions, and speed similarly showed increased values, supporting the hypothesis that FIP can initiate and maintain hyperreactivity, especially if the exposure period is prolonged. There was an increased number of counterclockwise rotations, suggestive of anxious behavior, in the treatment groups compared to the control group, and a lower amplitude of return angles [158].

Regarding the occurrence of anxiety, it was shown that avoidance of the dark zone by exposed fish and increased time spent in the light zone indicate an anxious state, as normally adult zebrafish tend to spend more time in the dark zone and prefer the light zone only in the larval stage. Depending on the dose, FIP and PYR influence locomotor activity and trigger anxious behavior in zebrafish. Both compounds act predominantly on the central nervous system, but the action is general and their interaction especially at the molecular level, should be explored and documented in future studies so that the results are fully reliable [158]. Wu et al. [164], studying acute exposure to FIP, showed a considerable decrease in the mean survival rate for exposed zebrafish, with the survival rate decreasing by 10% at 24–96 h after exposure. Beyond 96 h, the survival rate reached 50%. In fish treated with the highest dose of FIP, the average survival rate decreased to 10% after only 24 h. Basically, as the FIP concentration increases, the survival rate of fish gradually but significantly decreases [164]. Regarding the effects of FIP on zebrafish locomotion, fish in the treatment group had increased standing time and decreased average swimming speed directly proportional to the increased FIP concentration. Thus, while at the lowest dose, the effects were not significant, at the highest doses and longest exposure times, the mean speed and mean distance traveled decreased significantly. In other words, exposed zebrafish suffered numerous collisions or remained in the corners of the aquarium, suggesting that FIP may also produce anxiety-like behavior [164].

The effects of phenyl pyrazole compounds can also be observed histologically [187,188]. FIP has been reported to produce multiple tissue changes in different experimental animal models and to have a more pronounced effect on the central nervous system [83]. In common carp (*Cyprinus carpio*) exposed to FIP for 12 days, histopathological changes were identified in the gills, liver, kidney, and intestine [189]. Histological analysis after exposure of zebrafish to FIP + PYR revealed altered cell density (lower numbers of pericarions) in the most rostral region of the adult brain [158] and the changes observed per segment are listed in Table 3.

Neurons in the group of zebrafish exposed to 0.1 mg/L FIP + PYR exhibited a pyknotic nucleus and eosinophilic cytoplasm, indicating that the mixture at this concentration had an apoptotic effect [158]. Immunohistochemical staining and Western blot analysis showed increased TNF-α expression in treated fish. Therefore, it can be concluded that FIP can cause inflammation in the brain tissue of adult zebrafish [164]. The expression analysis of caspase-3, a marker protein for apoptosis, showed a significant increase in exposed fish, a result that was confirmed by both immunohistochemical staining and Western blot analysis, suggesting that FIP is responsible for promoting apoptosis in the brain tissue of adult zebrafish [164]. The expression of nuclear factor kappa-light-chain-enhancer of activated B cells (NF-kB) was also significantly increased in fish subjected to FIP treatment, and the protein expression of neuronal nuclei (NeuN) and brain-derived neurotrophic factor (BDNF) was significantly decreased [164]. These results suggest that FIP generates neurotoxicity in adult zebrafish, mainly due to oxidative stress, inflammation, and apoptosis in brain tissue. The changes observed in the histopathological analysis of treated zebrafish align with the changes observed in locomotor activity and the anxiety state, which may explain some of the behavioral findings [158]. In conclusion, FIP toxicity can cause damage to zebrafish brain tissue by inducing oxidative stress, inflammation, and apoptosis and can lead to impaired locomotion, affecting sensory and motor systems [164]. FIP significantly reduces the survival rate, and this is dose- and exposure-time-dependent [190].

## 5. Influence of Deltamethrin on Oxidative Stress Parameters and Behavioral Variables in Zebrafish

Deltamethrin (Del) is a commonly used pesticide in agriculture, forestry, aquaculture, and public health programs [191,192,193,194]. Since the use of organophosphorus and organochlorine pesticides has been restricted or banned due to their toxicity and residue-forming properties [195], pyrethroids have rapidly advanced to second place in sales in the global market because they have the advantages of high efficiency and low toxicity [196]. As a consequence of overuse, pyrethroid pesticides have been detected in various commodities, including crops, fruits, and vegetables [197,198], and pyrethroid residues have been detected in environments such as soil and surface water [199,200]. Del can be found in the form of crystals [201] or a crystalline white or beige powder [202] and is found In a variety of commercial preparations: sprayable powder, emulsifiable concentrate, emulsifiable granules, proprietary emulsion, pour-on solutions, fluid or soluble concentrates, tablets, water-dispersible granules, wettable powders, etc. [203]. The source of Del is its use as an insecticide for a wide range of crops, as a control substance for flying or crawling insects (domestic use), as a wood preservative, and as an external animal pest control agent. It is also used in public health programs, such as against Chagas disease and malaria, and to protect stored crops, especially cereal grains, coffee beans, and dry beans [203]. In recent years, the production and use of Del have undergone explosive growth [204]; it has been detected in over 50% of groundwater samples from rural areas in Iran [205] and in surface water and sediment, with concentrations ranging from 0.73 ng/L to 24 μg/L [206,207,208]. In addition, Del has been detected in human blood and urine samples [209].

In humans, skin contact with Del can lead to reddening of the skin or tingling at the application site, and if ingested or absorbed through the eyes, facial paresthesia is a common symptom, in addition to the general symptoms of pain, red and watery eyes, numbness of the tongue and lips, abdominal pain and vomiting. At high doses, signs of Del poisoning include profuse salivation, pulmonary edema, clonic convulsions, opisthotonos, coma, and even death [203].

Del temporally attacks the nervous system of any animal it comes in contact with. The general mechanism of both type I and type II pyrethroids is that they prolong the opening phase of sodium channel gates when nerve cells are excited. In insects, for example, Del acts by inducing paralysis through persistent inhibition of open sodium channels in the nervous system [210], thereby killing them. Pyrethroids have been shown to bind to the lipid phase of membranes in the immediate vicinity of sodium channels, thereby altering channel kinetics. This blocks the closure of sodium gates in nerves and thus prolongs the return of the membrane potential to the resting state. Repetitive neuronal discharge and prolonged negative potential produce effects similar to those produced by dichlorodiphenyltrichloroethane (DDT), leading to hyperactivity of the nervous system, which can lead to paralysis and subsequent death. Other mechanisms of action of pyrethroids include antagonism of GABA-mediated inhibition, modulation of nicotinic cholinergic transmission, increased noradrenaline release, and actions on calcium ions. They also inhibit Ca^2+^, Mg^2+^, and ATP-ase channels [203].

**Figure 3:**
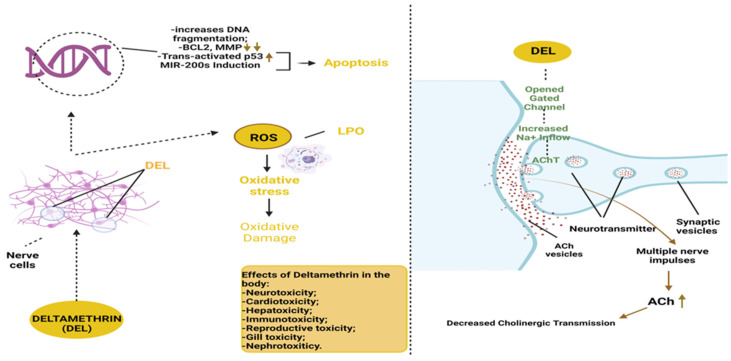
Mechanism of action of deltamethrin: Pyrethroids interfere with normal production and conduction of nerve signals in the nervous system. Type II pyrethroids, including deltamethrin, have an α-cyano group that induces long-lasting inhibition of sodium channel activation gates. This results in prolonged permeability of nerves to sodium and produces a series of repetitive nerve signals in sensory organs, sensory nerves, and muscles. Also, under the action of deltamethrin, oxidative stress and the apoptotic mechanism are triggered.

Thus, the mechanism of action of Del in the body is complex; it causes adverse effects on the nervous system [211] and is able to generate oxidative stress (Figure 3).

Although Del has been reported to be less toxic to mammals and birds [212], it has also been shown to be highly toxic to aquatic organisms [209], especially fish. Pyrethroid easily reaches aquatic ecosystems; once it is released into the environment, due to its hydrophobicity, it can adsorb onto any type of solid in suspension, causing significant disturbance to living organisms [213]. Research on the toxicological effects of Del has been carried out on several freshwater organisms, such as water fleas (*Daphnia magna*), snails (*Carassius carassius*) [214], rainbow trout (*Oncorhynchus mykiss*) [215,216], mirror carp (*Cyprinus carpio specularis*) [217] and zebrafish (*Danio rerio*) [218], in which the insecticide was found to cause significant damage. Exposure of zebrafish to Del triggered the following changes: AChE inhibition [219], altered swimming speed and depth [220], pericardial edema and neurotoxicity [221], delayed oogenesis [222], delayed embryonic development, larval malformations and reduced chorion surface tension [223].

Changes in behavioral and oxidative stress enzymes (SOD, CAT, MDA, GPx) after acute exposure to Del are shown in Table 4.

Huang et al. [220] measured the variable swimming speed of zebrafish exposed to Del and obtained slightly different values than those obtained by Strungaru et al. [224]. Huang et al. [220] recorded that after exposure to Del at a concentration of 0.15 µg/L (the lowest concentration used in the study) for 360 min, swimming speed increased from 39.6 to 49.7 mm/s. Swimming speed also increased when fish were exposed to the highest concentration in the study (15 µg/L), from 43 to 54.0 mm/s. However, these increases were only observed in the first 60–120 min [220], after which the values decreased, as in a study by Strungaru et al. [224].

Oxidative stress enzymes showed the following changes: malondialdehyde (MAD) increased, while SOD varied: it was the lowest in the group exposed to 25 µg/L, reflecting the presence of oxidative stress in the zebrafish body. However, higher SOD values were found for the group exposed to 12.5 µg/L than the control group, suggesting that Del has an antioxidant effect at this concentration [224].

CAT levels increased with increasing Del concentration in water, and GPx showed significantly lower values in the group exposed to 25 µg/L compared to the other groups.

An analysis of aggressive behavior showed that exposure to Del did not increase the level of aggression in zebrafish, suggesting that this behavioral change is not part of the response to insecticide toxicity [224], except in those exposed to 0.5–1 µg/L Del, in which aggressive behavior was triggered much earlier (only 2 days post-exposure). This is evidence that at low concentrations and with longer persistence in the environment, Del affects social interactions between fish, leading to mutual injuries, and this can be classified as a threat to fish species that depend on group integrity and cooperation to survive. In addition, fish exposed to 1 µg/L showed the largest number of alternations between aggressive and non-aggressive behavior, suggesting severe impairment of the cognitive part of the zebrafish brain.

Immunohistochemical aspects, revealed by the use of specific markers, showed fragmentation of nuclear DNA in apoptotic neurons in zebrafish exposed to Del, and different intensities of these markers were observed at the telencephalic level, specifically in the cells of the caudal dorsal area, in proportion to the increased Del concentration in the medium. Also, at this level, different cell densities in response to toxicity could be observed.

Staining for p53 protein and TUNEL was more intense in groups exposed to 1 and 2 µg/L of Del, and reduced staining of the proliferating cell nuclear antigen (PCNA) marker was decreased in telencephalon in groups exposed to the highest concentrations [224]. A proliferation index was also calculated with PCNA, and an increase was recorded, which indicates the self-protective action of the nervous system against insecticide toxicity. Similar results were obtained in the optic tectum and cerebellum. The scores obtained for quantitative intensity were also confirmed by immunohistochemistry results (IHC profiler analysis), showing that Del influenced the expression, calculated as energy/pixel unit for TIFF analysis, and the scores for the immunohistochemical profiler [224]. The histological and immunohistochemical results reveal that exposure to Del caused significant lesions in all brain areas analyzed (telencephalon, optic tectum, and cerebellum), and correlating the results of histological analysis with those obtained in the behavioral test, it can be seen that Del affects the cognitive function of fish.

## 6. Evaluation of Neurotoxicity after Exposure of Zebrafish to Different Doses of Rotenone

Rotenone (ROT) is a chemical compound commonly used in agriculture for pest control and in ecology to achieve beneficial control of wildlife in soil or water [225]. It is used practically as an insecticide and a pesticide, but is also used to exterminate certain fish populations; for example, in 1975, an attempt was made to eradicate invasive European carp from a lake in Tunisia using ROT [226]. ROT occurs as colorless to brownish crystals or as a white or brownish crystalline powder. It has neither taste nor odor [227]. It is found in several commercial forms, among which the emulsified concentrate is classified as highly toxic and can only be used by certified applicators [228]. ROT is also an isoflavone compound, which is naturally found in the jicama vine plant as well as many plants in the Fabaceae family. In the past, ROT was used to treat scabies and lice in humans and various ectoparasites of animals [229] and was later used to control mites in chickens and other birds, as well as lice and ticks that parasitize dogs and cats.

The sources of rotenone pollution are its use as a pesticide, piscicide, acaricide, and insecticide. Poisoning in humans, through accidental ingestion, can lead to convulsions and coma, and liver or kidney damage can occur. In the case of poisoning, the lethal oral dose was estimated to be 50 mg/kg, resulting in respiratory arrest and coma within 8 h of ingestion [230].

The insecticide is relatively easily degraded by environmental factors—air, water, and light [231,232]—and depending on the state of the formulation in which this active substance is found, it shows varying degrees of toxicity [232]. The use of ROT to control unwanted fish populations [232] has triggered imbalances in the biocenosis of the aquatic environment, showing that the substance is readily absorbed by fish and that they lack the enzymes necessary to degrade the compound. Thus, ROT causes toxicity, even if ingested in small quantities, and its action often leads to the death of aquatic organisms such as fish, amphibians, and even invertebrates [231,232]. ROT is easily absorbed into the body (there is evidence that it can cross the blood–brain barrier) due to its lipophilic state, and it accumulates mainly in the mitochondria [233]. Basically, the action of this substance inhibits complex I of the mitochondrial respiratory chain, which induces a decrease in ATP production and an increase in the amount of ROS, thus establishing oxidative stress [234,235]. The onset of oxidative stress will, over time, lead to the degradation of DNA, proteins, and lipids [236,237,238], creating a structural and functional imbalance in the body. In addition, ROT is able to significantly reduce the activity of glutathione, so that ROS will predominate in the body [239].

**Figure 4:**
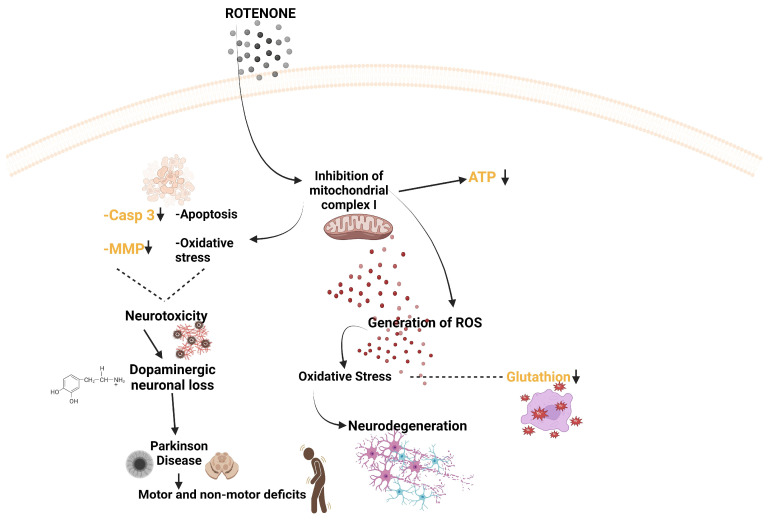
Exposure to rotenone causes neurotoxicity, manifesting as neurodegeneration, cell apoptosis, accumulation of fibrillary aggregates (containing α-synuclein protein) and oxidative stress, causing the appearance of reactive oxygen species (ROS) and a jump in glutathione levels. As far as the nervous system is concerned, all the effects caused by rotenone are comparable to symptoms specific to Parkinson’s disease (e.g., the presence of Lewy bodies, motor and postural impairments, and sensory and metabolic disorders). Rotenone’s main action is directed at the mitochondria, specifically complex I of the mitochondrial respiratory chain, which it inhibits, triggering all of the above. Figure was created with www.BioRender.com (accessed 19 January 2024).

In the insect organism, ROT inhibits the conversion of nicotinamide adenine dinucleotide (NADH) to energy, and the same action is evident in fish, amphibians, and mammals [230]. In addition, ROT has been shown to induce a loss of dopaminergic neurons with inhibition of mitochondrial complex I function [240]. Figure 4 schematically illustrates the mechanism of action of ROT in the organism.

Accidental or intentional exposure to ROT by humans or mammals causes a certain level of toxicity in the body [231,232], manifesting as the appearance of symptoms specific to Parkinson’s disease [239]. Specific symptoms usually appear when the body is exposed to large amounts of the substance. Parkinson’s disease is responsible for irreversible and unidirectional degeneration of dopaminergic neurons. The consequences of this degeneration include inhibition of dopamine synthesis, followed by the appearance of fibrillar aggregates, called Lewy bodies, in the structure of which the protein α-synuclein is found [241]. The clinical picture of PD is a specific one and includes bradykinesia, essential tremor, rigidity, and postural imbalance. Non-motor symptoms such as sensory, gastrointestinal, and sleep disturbances are also present in the disease [242].

In the brain of adult zebrafish, dopaminergic neurons project to the ventral telencephalon and are located in the posterior tubercle of the ventral diencephalon [243]. The dopaminergic system in embryonic zebrafish is well characterized, with dopaminergic neurons first detected at 18 hpf in the ventral diencephalon; by 72 hpf, the organization of the central nervous system is complete, and its further development increases the number of dopaminergic neurons [244,245]. Zebrafish dopaminergic neurons are also found in the olfactory bulb, preoptic region, retina, and pretectum [246]. In addition, fish have been found to have dopaminergic signaling pathways similar to mammals, and transcription factors have been shown to participate in the development of dopaminergic neurons in zebrafish [243,247]. Due to advances in the use of zebrafish, the effects of ROT have been elucidated, and it has been proven that it is a substance capable of producing motor dysfunction [248] similar to that found in PD, which progressively affects the nervous system [249]. Research has also been carried out on rats, in which effects such as hypokinesia and muscle rigidity have been observed following the exposure of animals to ROT, and studies in mice and *Drosophila* have illustrated symptoms similar to those seen in PD [248,250]. After acute exposure to ROT, the locomotor activity of zebrafish was found to be not altered, with similar values for swimming activity, range of motion, freezing duration, and distance traveled during freezing for fish in the control and treatment groups [251]. However, after a longer ROT treatment of 4 weeks, fish showed reduced swimming duration and distance traveled compared to those in the control group [251], suggesting that prolonged exposure impairs motor capacity, inducing bradykinesia. Khotimah et al. [252] demonstrated that subchronic exposure (28 days) to 5 µg/L of rotenone caused parkinsonism due to decreased dopamine levels, locomotor activity, increased α-synuclein expression and aggregation, and apoptosis of dopaminergic neurons. Decreased locomotor activity in zebrafish is thought to be due to mitochondrial dysfunction, causing reduced ATP production, disruption of mitochondrial permeability, and increased Ca^2+^ and ROS overproduction. These conditions can lead to autooxidation of dopamine or its enzyme, tyrosine hydroxylase, thereby reducing dopamine as a neurotransmitter for motility. Given the locomotor dysfunction seen in fish after prolonged exposure to ROT, an association can be made between the administration of the pesticide and the development of PD, as this clinical manifestation is prevalent in the pathology [249,253] and reflects the loss of dopaminergic neurons.

Depression is one of the most common non-motor symptoms of PD and is often associated with anxiety [253,254]. Anxiety analysis using the light/dark test indicated that ROT-treated fish spent more time in the light compartment and exhibited significant latency in the dark compartment entry zone. These phenomena demonstrate that ROT induces anxiety- and depression-like behavior in zebrafish [251].

After exposure to ROT, levels of dopamine (DA), dihydroxy-phenylacetic acid (DOPAC), 5-hydroxytryptamine (5-HT), 5-hydroxyindoleacetic acid (5-HIAA), and norepinephrine (NE) were measured [251]. Fish in the treatment group were found to have a 40% decrease in brain DA levels. They also had an increased DOPAC/DA ratio, but no significant change in DOPAC levels was evident. Levels of the other neurotransmitters analyzed did not change.

The decrease in DA revealed the association of ROT with PD mechanisms, since in this disease dopaminergic neurons are lost and, implicitly, dopamine is depleted [249], leading to the specific symptomatology. Another distinctive sign of PD is oxidative stress [253,255], and it has been shown that the underlying mechanism of GI dysfunction in this disease is an imbalance between oxidants and antioxidants in the gut and brain. The explanation for the mechanism is that inflammation in the gut can cause systemic inflammation, and proinflammatory cytokines such as interleukins IL-1, IL-6, and IL-21, TNF-α and interferon gamma (IFN-γ) can enter the brain, either via the microbiota–gut–brain axis or blood–brain barrier permeability [256,257]. These proinflammatory cytokines stimulate glial cells to produce other inflammatory factors, ROS, and reactive nitrogen, inducing more inflammatory reactions and leading to neuroinflammation and consequent neurodegeneration [250,257]. Dopaminergic neurons in zebrafish have been shown to be sensitive to oxidative stress [258,259]. In one study, zebrafish were exposed to 2 mg/L ROT for 4 weeks and the locomotor activity of the fish was followed. In fish exposed to ROT, there was decreased locomotor activity, evidenced by a decrease in total distance traveled by the fish, and oxidative stress enzymes showed increased LPO and decreased GST and CAT in both brain tissue and gut. An increased level of NO was recorded only in the brain, and SOD activity showed a slight increase in the brain tissues of fish exposed to ROT and a decrease in the gut [260].

Ilie et al. [241] found that ROT did not significantly influence locomotor activity, with fish maintaining their exploratory behavior throughout the period analyzed [241]. However, the oxidative stress parameters measured showed decreased SOD and malondialdehyde (MDA) in the ROT group, but the values did not reveal a marked difference compared to the control group. While the levels of LPO, MDA and SOD were increased after treatment of fish with ROT in a study by Unal et al. [260], in Ilie’s experiment, the SOD and MDA levels were lower, but this is explained by the fact that in Ilie et al.’s experiment [241], oxidative stress markers were evaluated for the whole fish, not only for the brain and gut [241], and the exposure doses were different (2.5 µg/L and 2000 µg/L, respectively).

Histologically, moderate labeling was evident in the optic tectum based on the immunohistochemical markers used. PCNA labeled two small areas of neural stem cells (NSCs) and neuroblasts [241]. PCNA, S100b, GFAP, and cox4i1 markers showed intense expression in the torus longitudinalis, torus semicircularis, and basal tegmentum, and S100b protein immunoreactivity was detected in the optic tectum, expressively labeling nerve fibers.

In the ROT-exposed group, there was a reduction, even an absence, of PCNA, GFAP, and S100, as well as moderate labeling for p53 and cox4i1. This reduction in PCNA labeling suggests a decrease in neurogenesis and an increase in neuronal dysfunction, especially as it is associated with a reduction in GFAP and S100b [241]. However, p53 and cox4i1 expression in the ROT group indicates aspects of mitochondrial dysfunction and apoptosis that are also found in the PD mechanism.

A predominance of radial glial cells and gray matter neurons in the molecular, Purkinje, and granular layers of the cerebellum was observed. These were labeled with S100b, p53, GFAP, and cox4i1 with an intensity close to that found in the optic tectum. The S100b protein has been found in the cerebellum, particularly in small neurons in the superficial molecular layer [241]. ROT has a toxicological profile that makes it capable of triggering symptomatology similar to that found in Parkinson’s disease, but the toxicity is dose- and time-dependent.

Although the pesticides described (fipronil, deltamethrin, and rotenone) have distinct chemical structures and modes of action, they may share some commonalities in terms of their potential environmental impact, such as toxicity to non-target organisms, bioaccumulation in food chains and persistence in the environment. Additionally, they may be regulated and managed similarly in terms of pesticide registration, usage restrictions, and environmental monitoring. However, it is important to recognize the differences between these pesticides as well as their specific properties and effects when considering their use and regulation.

In summary, fipronil, deltamethrin, and rotenone are pesticides that can have neurotoxic effects on zebrafish through different molecular targets and pathways. These neurotoxic effects can manifest as alterations in swimming behavior, locomotor activity, motor coordination, and neuronal integrity, highlighting the vulnerability of zebrafish to pesticide exposure and the importance of assessing the ecological impacts of pesticide contamination in aquatic ecosystems.

## 7. Conclusions

Microplastics, nanoplastics, and pesticides are interconnected environmental pollutants that can have detrimental effects on zebrafish and other aquatic organisms. The link between microplastics, nanoplastics, and pesticides in the aquatic environment lies in their common ability to coexist and interact with each other, leading to complex ecological consequences. They are interconnected and can produce cumulative effects on zebrafish, as follows:

- Co-occurrence and sorption: Microplastics and nanoplastics can serve as carriers of or sorbents for pesticides in aquatic environments. Pesticides can adsorb onto the surface of plastic particles, leading to their accumulation and persistence in the water column, sediments, and biota. This co-occurrence increases the exposure of aquatic organisms, including zebrafish, to both plastic pollution and pesticide contamination.
- Bioaccumulation and trophic transfer: Microplastics, nanoplastics, and pesticides can bioaccumulate and trophically transfer in aquatic food webs. Zebrafish can ingest plastic particles and pesticides directly or indirectly through their diet, leading to the accumulation of these contaminants in their tissues over time. Bioaccumulation and trophic transfer can amplify the concentrations of microplastics, nanoplastics, and pesticides at higher trophic levels, including in zebrafish predators, further exacerbating their ecological impact.
- Synergistic effects and toxicity: Microplastics, nanoplastics, and pesticides can have synergistic or additive effects on zebrafish and other aquatic organisms. Combined exposure to plastic particles and pesticides can increase the toxicity of individual contaminants, leading to greater adverse effects on the health and physiology of zebrafish. Synergistic effects can arise from interactions between plastic-induced stress responses, such as inflammation and oxidative stress, and pesticide-induced neurotoxicity, developmental toxicity, or endocrine disruption in zebrafish.

Overall, the simultaneous occurrence and interaction of microplastics, nanoplastics, and pesticides in the aquatic environment poses significant ecological risks to zebrafish and other aquatic organisms. Understanding the linkages between these pollutants as well as their impacts is crucial for mitigating environmental contamination and protecting aquatic ecosystems.

## Figures and Tables

**Table 1:** Behavioral and metabolic changes generated by FIP in zebrafish.

Doses (mg/L)	Exposure Time	Effects	Authors
0.5, 1 and 2	96 h	Increased SOD and CAT activity Increased lipid peroxidation	[164]
2.5, 7.5 and 15	72 h	Decreased cell proliferation	[184]
0.4 and 0.8	5 days	Increased anxiety Disturbance of swimming behavior Increased lipid peroxidation	[178]
0.33 and 0.8	5 days	Locomotor defects	[175]

**Table 2:** Behavioral and metabolic changes induced by PYR in zebrafish.

Doses (mg/L) and Exposure Times	Effects	Authors
0.0765 and 21.461	Inhibited AChE activity Increased ROS levels	[183]
0.125, 0.675 and 1.75 (96 h)	No locomotor disorders recorded No anxiety-like behaviors observed	[185]
1.66 (96 h)	Increased ROS levels Increased lipid peroxidation Increased nitric oxide levels Decreased SOD, CAT, GPx levels	[186]

**Table 3:** Histological changes revealed after exposure of zebrafish to FIP + PYR.

Area Analyzed	Histological Changes	Author
Telencephalon	Increased number of blood vessels (some being ectatic) and blood cell infiltration in both treatment groups	[158]
Diencephalon and Mesencephalon	Dilation of blood vessels and leukocyte infiltration in both treatment groups; central chromatolysis distinguished in large neurons in the oculomotor nucleus	
Rhombencephalon	Mild infiltration and neuronal damage were evident, especially in group of fish exposed to highest concentration	
Spinal cord	Only edema of pericardium was observed in some motor neurons, and intense vascularization	
Cerebellum	No obvious changes were observed	

**Table 4:** Changes in swimming behavior of zebrafish after exposure to different doses of deltamethrin.

Behavioral Parameters Measured	Dose of Exposure (µg/L)	Results Recorded in Pre-Treatment Group	Results Recorded 2 h PostExposure	Effects	Author
Total swimming distance	DM 25	791.6 ± 264.9 cm	337.9 ± 218.6 cm	Total swimming distance decreased post-exposure in both experimental groups	[224]
	DM 12.5	721.3 ± 259.7 cm	251 ± 137 cm		
Variable swim velocity	DM 25	3.2 ± 1.2 cm/s	1.06 ± 0.56 cm/s	Variable swimming speed decreased significantly	
	DM 12.5	3.03 ± 1.07 cm/s	1.06 ± 0.56 cm/s		
Active swimming	DM 25	215.7 ± 35.17 s	157.17 ± 57.79 s	Zebrafish exposed to these concentrations showed lethargic behavior and became less active	
	DM 12.5	216.3 ± 49.2 s	159.4 ± 57.7 s		
Counterclockwise rotations	DM 25	5.25 ± 4.2	1.87 ± 1.12	Counterclockwise movement decreased significantly	
	DM 12.5	4.73 ± 3.53	2 ± 1.6		
Clockwise rotations	DM 25	5.13 ± 2.2	3 ± 3	Clockwise revolutions decreased significantly	
	DM 12.5	6.7 ± 4.3	1.6 ± 0.9		
